# Odor-Induced Multi-Level Inhibitory Maps in *Drosophila*

**DOI:** 10.1523/ENEURO.0213-19.2019

**Published:** 2020-01-09

**Authors:** Veit Grabe, Marco Schubert, Martin Strube-Bloss, Anja Reinert, Silke Trautheim, Sofia Lavista-Llanos, André Fiala, Bill S. Hansson, Silke Sachse

**Affiliations:** 1 Department of Evolutionary Neuroethology, Max Planck Institute for Chemical Ecology, Jena 07745, Germany; 2 Department of Molecular Neurobiology of Behavior, Johann-Friedrich-Blumenbach-Institute for Zoology and Anthropology, University of Göttingen, Göttingen 37077, Germany

**Keywords:** antennal lobe, chloride imaging, *Drosophila*, inhibition, olfactory coding, sensory processing

## Abstract

Optical imaging of intracellular Ca^2+^ influx as a correlate of neuronal excitation represents a standard technique for visualizing spatiotemporal activity of neuronal networks. However, the information-processing properties of single neurons and neuronal circuits likewise involve inhibition of neuronal membrane potential. Here, we report spatially resolved optical imaging of odor-evoked inhibitory patterns in the olfactory circuitry of *Drosophila* using a genetically encoded fluorescent Cl^-^ sensor. In combination with the excitatory component reflected by intracellular Ca^2+^ dynamics, we present a comprehensive functional map of both odor-evoked neuronal activation and inhibition at different levels of olfactory processing. We demonstrate that odor-evoked inhibition carried by Cl^-^ influx is present both in sensory neurons and second-order projection neurons (PNs), and is characterized by stereotypic, odor-specific patterns. Cl^-^-mediated inhibition features distinct dynamics in different neuronal populations. Our data support a dual role of inhibitory neurons in the olfactory system: global gain control across the neuronal circuitry and glomerulus-specific inhibition to enhance neuronal information processing.

## Significance Statement

Neural inhibition is evidently as important as excitation given it is present at every level of sensory processing. This study characterizes odor-evoked inhibitory patterns along different levels of olfactory processing of *Drosophila* using functional imaging via Clomeleon, a genetically encoded indicator for chloride ions, the main mediator of synaptic inhibition in mature neurons. In combination with the excitatory component reflected by intracellular calcium, we analyzed the interplay between odor-evoked excitation and inhibition. Our data provide both a more accurate and comprehensive characterization of the actual information content encoded by the olfactory circuitry, as well as elucidate network properties within the primary olfactory center of the fly.

## Introduction

Inhibition of neural excitability is a ubiquitous feature of all neuronal circuits. Neurons that release inhibitory transmitters are present in all parts of the nervous system. In the olfactory systems of both insects and vertebrates, inhibition is crucial for stimulus gain control (Olsen and Wilson, 2008; Root et al., 2008), synchronizing neural networks (Laurent et al., 2001), generating precise timing (Schoppa and Westbrook, 1999; Margrie and Schaefer, 2003), broadening transmission of olfactory signals (Nagel et al., 2015), odor mixture interactions (Mohamed et al., 2019), and enhancing contrast between similar odor representations (Mori et al., 1999; Sachse and Galizia, 2002; Urban, 2002). In the mammalian olfactory bulb, inhibition is largely mediated by dendrodendritic synaptic connections between excitatory mitral cells and inhibitory granule cells (Schoppa and Urban, 2003; Egger and Urban, 2006). Despite these important roles of inhibition for odor processing, most studies analyzing olfactory coding at the level of spatially distributed neuronal populations focused on monitoring neuronal excitation. Therefore, odor representations at the level of the insect antennal lobe (AL) or the vertebrate olfactory bulb typically have been characterized as patterns of excitation. Here, we aimed at monitoring spatially distributed maps of odor-evoked inhibition at different levels of processing in *Drosophila melanogaster*.

In the fly, odors are detected by olfactory sensory neurons (OSNs) located on the antennae and maxillary palps. Each OSN typically expresses one or very few chemo-receptor genes, and each OSN projects its axon to the AL, the insect analog of the vertebrate olfactory bulb. In the AL, those OSNs expressing the same odorant receptor (OR) stereotypically converge to the same spatially invariant olfactory glomeruli (Couto et al., 2005; Fishilevich and Vosshall, 2005), each of which can be unambiguously identified (Laissue et al., 1999; Grabe et al., 2015). The AL is densely innervated by local interneurons (LNs) that mediate both intraglomerular and transglomerular inhibition (Wilson and Laurent, 2005; Seki et al., 2010). Olfactory projection neurons (PNs) convey the olfactory signals to higher-order brain centers.

The morphologic structure of the AL network specifies the physiologic logic of how odors are encoded: Each odorant evokes a characteristic, spatiotemporal activity pattern leading to a combinatorial, stereotypic activation of glomeruli in the AL (Fiala et al., 2002; Wang et al., 2003). Inhibitory LNs provide both feedforward synaptic inhibition of PNs and feedback inhibition of OSNs (Wilson and Mainen, 2006; Olsen and Wilson, 2008; Root et al., 2008). However, it still remains elusive how spatially distributed, odor-evoked inhibition interferes with and relates to the well-described excitation-based odor maps.

In *Drosophila*, functional imaging has mainly relied on genetically expressed Ca^2+^ sensors that detect intracellular Ca^2+^ dynamics as a correlate of neuronal excitation (Grienberger and Konnerth, 2012). In this study, we monitored odor-induced inhibitory maps in the olfactory circuitry using a DNA-encoded indicator for Cl^-^, the main ionic mediator of synaptic inhibition in mature neurons (Owens and Kriegstein, 2002). The FRET-based indicator Clomeleon consists of a Cl^-^-sensitive yellow fluorescent protein (YFP) and a Cl^-^-insensitive cyan fluorescent protein (CFP; Kuner and Augustine, 2000). Binding of Cl^-^ to YFP reduces its absorbance, which results in a change of the YFP/CFP emission ratio proportional to [Cl^-^]_i_. The applicability of Clomeleon *in vivo* has been demonstrated in hippocampal slices (Berglund et al., 2006), retinal bipolar cells (Haverkamp et al., 2005; Duebel et al., 2006), thalamo-cortical neurons of mice (Glykys et al., 2009) and cerebellar granule cells (Berglund et al., 2016).

We genetically expressed Clomeleon in defined olfactory neurons and characterized odor-evoked inhibition at different levels of olfactory processing in comparison with Ca^2+^-mediated activity using the likewise FRET-based Ca^2+^-sensitive protein Cameleon 2.1 (Miyawaki et al., 1999). First, we observed odor-evoked Cl^-^-influx in dendrites of OSNs. Second, we generated a comprehensive functional map of both odor-evoked activation and inhibition of the fly AL. We demonstrate that odor-evoked inhibition carried by Cl^-^ influx is characterized by stereotypic odor-specific patterns. Third, we show that Cl^-^-mediated inhibition exhibits distinct features at different levels of olfactory processing pointing toward multiple roles of inhibition in the olfactory system.

## Materials and Methods

### *Drosophila* stocks and *in vivo* preparation

All fly stocks were maintained on conventional cornmeal-agar-molasses medium under 12/12 h light/dark conditions, relative humidity of 70% and at 25°C. The Clomeleon DNA construct (Kuner and Augustine, 2000), kindly provided by Thomas Kuner, was inserted into the pUAST vector (Brand and Perrimon, 1993) via the EcoRI and XhoI restriction sites. Transgenic constructs were injected by Genetic Services Inc. into *yw* embryos using standard procedures and single transformants were outcrossed to autosomal balancers for chromosomal mapping. Two independent insertions on different chromosomes were combined. Homozygous female flies, 6–10 d old, carrying four copies of the UAS:Clomeleon transgenes, were used for all imaging experiments. The fly strain *UAS-Cameleon 2.1* (Fiala et al., 2002) was chosen for monitoring odor-evoked Ca^2+^ signals as an appropriate FRET-based sensor comparable in its chromophores with Clomeleon. *Orco-Gal4* (RRID:BDSC_23292; Wang et al., 2003), *Or22a-Gal4* (RRID:BDSC_9951; Vosshall et al., 2000), and *GH146-Gal4* (RRID:BDSC_30026; Stocker et al., 1997) were used to drive expression of *UAS-Clomeleon* or *UAS-Cameleon* (RRID:BDSC_6901).

### Optical imaging

For imaging intracellular Cl^-^ and Ca^2+^ dynamics in the AL, flies were restrained in custom-built holders and a small window was cut into the head capsule. The hole was covered with physiologic saline solution, and imaging was performed using a water immersion objective directly positioned above the exposed brain. Pharmaca [GABA, potassium gluconate (KGlu), PTX, 5-nitro-2(-3-phenylpropylamine) benzoic acid (NPPB)] were applied by exchanging the saline drop on the brain by a drop of the approximate volume and the targeted concentration. For NPPB an additional ethanol application was conducted to control for the solvent effect (data not shown). For transcuticular antennal imaging, flies were restrained as for the *in vivo* dissection method without opening the head capsule. Or22a-expressing OSNs were imaged from the posterior side of the antenna, while the majority of OSNs using *Orco-Gal4* were recorded from the anterior side.

Imaging experiments were performed using TillPhotonics imaging equipment (TILL imago, Till Photonics GmbH) with a CCD-camera (PCO imaging, Sensicam) and a fluorescence microscope (Olympus, BX51WI) equipped with a 20× water immersion objective (NA 0.95, XLUM Plan FI, Olympus) for AL imaging and a 10× air objective (NA 0.30, UPlan FLN, Olympus) for antennal imaging. A monochromator (Polychrome V, Till Photonics) provided light at 440 nm excitation wavelength which was guided through a 470-nm dichroic short pass filter. The beam-splitter (Optical Insights, DV-CC) separated YFP from CFP emission with a 505 DCXR and narrowed the emissions with bandpass filters of 535/30 nm for YFP and 465/30 nm for CFP. Images of both emitted wavelengths were projected side by side onto a single CCD camera chip (PCO Imaging, Sensicam). Fourfold binning on the CCD-camera chip resulted in an image size of 344 × 260 pixels with one pixel corresponding to 1.25 × 1.25 μm. Each recording lasted for 20 s with an acquisition rate of 2 Hz. Since Clomeleon yielded a very low signal-to-noise ratio, we had to apply long exposure times which limited our recording frequency. We also performed experiments with the usually used frequency of 4 Hz, resulting in weaker signal intensities and a lower dynamic range. Since these signals did not reveal different temporal patterns in the odor-evoked responses as the slower recorded signals, we decided in favor of an increased signal-to-noise ratio and maintained a recording frequency of 2 Hz for the whole study. Odors were applied 2 s after experiment onset and lasted for 2 s. Individual flies were imaged for up to 1 h, with interstimulus time intervals of 1–3 min.

### Odor stimulation

Pure odorants were diluted in mineral oil (BioChemika Ultra; odor CAS: ethyl-3-hydroxybuytrate: 5405-41-4, benzaldehyde: 100-52-7, acetic acid: 64-19-7, cis-vaccenylacetate: 6186-98-7, pentyl acetate: 628-63-7, 1-hexanol: 111-27-3, ethyl benzoate: 93-89-0). For use, 6 μl of 1:10 diluted odor was pipetted onto a small piece of filter paper (100 mm^2^, Whatman), which was inserted into a glass Pasteur pipette. A stimulus controller (Syntech, Stimulus Controller CS-55) was used to apply the odor into a continuous airstream at 1 l/min, monitored by a flow meter (Cole Parmer). An acrylic glass tube guided the airflow to the fly antennae. Two additional air sources (airflow 0.5 l/min) were connected to the tube and the stimulus controller. One of them consisted of the glass pipette containing the odor on filter paper and was hooked up for odor application, whereas the other pipette was empty and added clean air to the continuous airstream forming an air equation which was switched off during odor application.

### Data analysis

Data were analyzed using custom-written IDL software (ITT Visual Information Solutions). First, a rigid registration was accomplished for all recordings separately to minimize movement artifacts throughout the time series. Second, the ratio of the two fluorescent signals was calculated as F_YFP_/F_CFP_ for every time point. The ratio (R) represents the relative magnitude of the signal intensity. To achieve a comparable standard for the calculation of the relative fluorescence changes of the ratio (ΔR/R), the fluorescence background was subtracted from the averaged values of frames 0–5 in each measurement, such that baseline fluorescence was normalized to zero. The false color-coded fluorescence changes in the raw-data images were calculated as the delta of frame 5 and 30 (Clomeleon) and frame 5 and 15 (Cameleon). Specific time traces of a measurement depict the mean of a 7 × 7-pixel coordinate (i.e., 9 × 9 μm), which was positioned into an anatomically identified glomerulus and plotted as a function over time. Since *GH146-Gal4* does not label glomeruli VM5d and VM5v, these could not be characterized at the PN level (Grabe et al., 2015). To generate schematic AL maps, the mean value of frames 10–30 for Clomeleon and 10–15 for Cameleon of a specific glomerulus and odor was averaged over all animals imaged. Although the chloride and calcium kinetics are clearly odor induced, they develop very slowly over time and show their maximal response change after odor offset. We therefore selected a delayed time window for our signal evaluation to capture the maximum/minimum of the odor-induced responses. One has to keep in mind that the monitored Ca^2+^ and Cl^-^ dynamics are also dependent on the kinetics and concentrations (i.e., expression levels) of the fluorescent sensors and might not reflect accurately the physiologic time traces. However, this issue is more relevant for fast stimulus dynamics (Martelli and Fiala, 2019), while with regard to slow recording frequencies, as used here, the resulting kinetics of Cl^-^ and Ca^2+^ binding are rather negligible.

Responses in each fly were normalized to the highest Cl^-^ or Ca^2+^ signal in each animal over all odors. To extract the temporal aspect of odor separation in the different neuronal populations, Euclidean distances (*L*2-Norm) were calculated. To compare the results, we always used the same set of 11 identified glomeruli in each data set. For a given stimulus *a*, the n-dimensional population vector (*v^a^*) was constructed using the relative fluorescence changes over time. Then the population vectors of two stimuli, *a* and *b*, were used to calculated the distance for every single data point (time) in the 40 frames as follows: *d(t) = (Σ(v_i_^a^(t) – v_i_^b^(t))^2^)^1/2^*, where *i* is an index for the i-th glomerulus. In addition to the Euclidean distances, principal component analysis was used to visualize the population activity of OSNs and PNs depending on the imaged reporter protein. Population vectors of all odor stimulations were aligned, taking into account time as the source of sample points, and number of glomeruli as the dimension of the original component space using the MATLAB statistical toolbox. All statistical analyses were performed using GraphPad InStat 3 as specified in each figure legend.

## Results

### Clomeleon as an indicator of intracellular Cl^-^ dynamics in olfactory neurons

**Figure 1.**
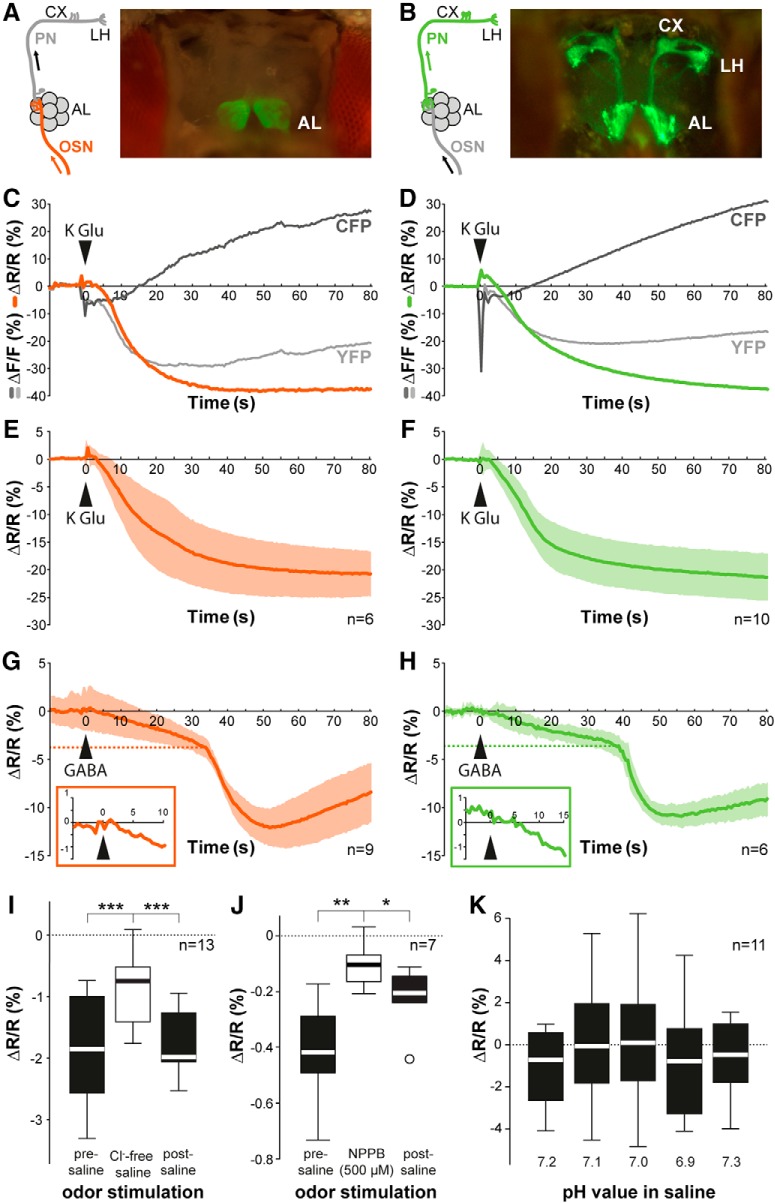
Clomeleon is a functional chloride indicator in *Drosophila* olfactory neurons. ***A***, ***B***, Schematic of AL neurons indicating expression site of Clomeleon with images of brain preparation showing Clomeleon YFP baseline fluorescence (***A***, OSNs; ***B***, PNs). AL, antennal lobe; CX, calyx; LH, lateral horn. ***C***, ***D***, Fluorescence change in a representative animal over time of CFP, YFP, and YFP:CFP ratio induced by KGlu application (1 M, 20 μl) into saline (300 μl) in OSNs (***C***) and PNs (***D***). ***E***, ***F***, Time course of [Cl^-^]_i_ increase induced by applying KGlu (arrowhead) averaged across several animals in OSNs (***E***) and PNs (***F***). Color shading indicates SD, *n* = 6–10. ***G***, ***H***, Time courses of [Cl^-^]_i_ increase induced by GABA application (1 M, 20 μl) into saline (300 μl) averaged across several animals in OSNs (***G***) and PNs (***H***). Insets show enlarged area around the time point of GABA application. Dashed lines mark the biphasic response threshold. Color shading indicates SD, *n* = 6–9. ***I***, Effect of Cl^-^ free saline application on Cl^-^ changes evoked by ethyl-3-hydroxybutyrate in OSNs. Box plots represent median value (horizontal line inside the box), interquartile range (box), and minimum/maximum value (whiskers). Removing Cl^-^ significantly reduced the Clomeleon signal (***p < 0.001, repeated measures ANOVA, *n* = 13). ***J***, Effect of chloride channel blocker NPPB (500 μM) application on Cl^-^ signals evoked by ethyl-3-hydroxybutyrate in OSNs (**p < 0.01, *p < 0.05, repeated measures ANOVA, *n* = 7). ***K***, Quantification of Clomeleon baseline fluorescence in OSNs at different saline pH in relation to standard condition (i.e., pH 7.3). Arrangement of different box plots from left to right reflects temporal sequence of the experiment (*p* = 0.144, repeated measures ANOVA, *n* = 11).

**Figure 2.**
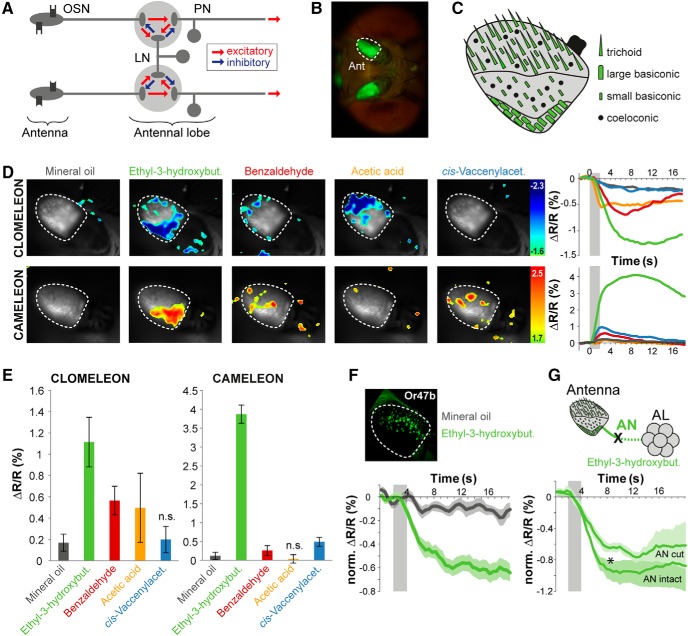
Odor application induces spatially confined chloride influx in OSNs in the *Drosophila* antenna. ***A***, Network model scheme of neuronal connectivity in the fly AL. ***B***, Clomeleon YFP baseline fluorescence in OSN dendrites in the *Drosophila* antenna (Ant). ***C***, Schematic of olfactory sensilla distribution on the third antennal segment. Sensilla marked in green are labeled by *Orco-Gal4*. ***D***, Pseudocolor rendering of odor-evoked changes in Cl^-^ concentration using Clomeleon (upper row) and in Ca^2+^ concentration using Cameleon (lower row) in response to different odors and mineral oil in OSN in the antenna. Images represent ΔR/R (%) superimposed onto raw fluorescence images according to the scales and color codes on the right. Time courses on the right reveal representative Cl^-^ and Ca^2+^ signals for different odors across the entire antennal segment. Odor application is indicated by a gray bar. ***E***, Quantification of Cl^-^ (left) and Ca^2+^ (right) responses to different odors and mineral oil (n.s., not significant from solvent; repeated measures ANOVA followed by Dunnett multiple comparisons test, *n* = 8). ***F***, Maximum intensity projection of Clomeleon expressed in the antenna under the control of *Or47b-Gal4*. Time courses of normalized Cl^-^ responses to the solvent control mineral oil and ethyl-3-hydroxybutyrate (*n* = 8). ***G***, Schematic of the connection between the antenna and AL via the antennal nerve (AN), which was disrupted here. Time course of normalized Cl^-^ responses to ethyl-3-hydroxybutyrate in three animals of the left antenna (AN intact, solid line) and right antenna after the right AN was cut (AN cut, dotted line). Lines indicate means, color shading gives SEM (*p < 0.05, two-way ANOVA).

We generated flies carrying the genetically encoded Cl^-^ sensor Clomeleon (Kuner and Augustine, 2000) to visualize *in vivo* Cl^-^-mediated inhibitory responses in the olfactory system. Using the binary GAL4-UAS transcriptional system (Brand and Perrimon, 1993), we expressed Clomeleon in the majority of OSNs using *Orco-Gal4* (Wang et al., 2003) and in PNs using *GH146-Gal4* (Stocker et al., 1997; Fig. 1*A*,*B*). To test whether Clomeleon is functional in *Drosophila* olfactory neurons, we optically monitored fluorescence changes in OSNs and PNs in the AL, while we applied KGlu to induce neuronal excitation globally and, concomitantly, inhibition through LN input onto OSNs and PNs (Fig. 1*C–F*; see network scheme in Fig. 2*A*). Applying KGlu increased CFP fluorescence, while YFP fluorescence was strongly decreased; thereby, the YFP/CFP ratio was reduced. To verify that this reflected inhibition, we applied the inhibitory transmitter GABA. GABA application immediately reduced the YFP/CFP ratio in both OSNs and PNs (Fig. 1*G*,*H*). Notably, we observed a second, strong emission decrease which was delayed by ∼35 s. The source of this second decrease is yet unclear, but could be due to the slow diffusion rate of GABA as it is bath applied to the whole brain and not actively perfused. The gradually increasing GABA concentration might surpass a threshold that initiates a strong inhibition reflected by the second phase. In combination with a gradual desensitization toward GABA (Hong and Wilson, 2015), this could explain the observed slow and biphasic GABA effect. To confirm that these ratio changes were dependent on Cl^-^, we removed Cl^-^ from the saline solution covering the fly’s brain. Odor application before Cl^-^ removal induced a clear ratio change, which was significantly reduced using Cl^-^-free saline (Fig. 1*I*). To further verify that our reporter was reflecting the intracellular Cl^-^ concentration, we applied the chloride channel blocker NPPB to block Cl^-^ channels in *Drosophila* neurons (O’Donnell et al., 1998). As expected, application of NPPB strongly reduced the Cl^-^ influx which was partly reversibly (Fig. 1*J*).

Since the YFP fluorescence has been reported to be affected by the pH value at [Cl^-^]_i_ above 50 mM (Kuner and Augustine, 2000), we verified that the fluorescence emission was not influenced by pH changes within the physiologically relevant range of 6.9–7.3 (Fig. 1*K*). This is in accordance with the described [Cl^-^]_i_ in OSNs, which is ∼24 mM in moths (Steinbrecht, 1992) and ∼20 mM in flies (Reinert et al., 2011). Therefore, a potential influence of pH changes on Clomeleon is negligible. Overall, our results confirm that Clomeleon functions reliably as a Cl^-^ indicator in olfactory neurons of the *Drosophila* AL.

### Odor stimulation induces peripheral Cl^-^ influx in dendrites of OSNs

Next, we analyzed whether odor stimulation causes a Cl^-^ increase at the most peripheral level of sensory transduction and performed transcuticular Cl^-^ imaging in OSN dendrites located on the fly’s antenna (Fig. 2*A*,*B*). Odor stimulation induced an odor-specific, spatially confined increase in [Cl^-^]_i_. These spatially restricted signals correspond to distinct sensillum types, which have well-described, specific distributions on the third antennal segment (Shanbhag et al., 1999; Grabe et al., 2016; Fig. 2*C*,*D*). To determine which sensillum types were excited by the odors used, we performed Ca^2+^ imaging in comparison using the ratiometric Ca^2+^ indicator Cameleon 2.1 (Miyawaki et al., 1999). Cl^-^ signals are characterized by a reduction in the Clomeleon’s YFP/CFP ratio (= increase in [Cl^-^]_i_), whereas Ca^2+^ signals are indicated by a ratio increase in the Cameleon’s YFP/CFP ratio (= increase in [Ca^2+^]_i_; Fig. 2*D*). Notably, some odors, such as ethyl-3-hydroxybutyrate, evoked both a Ca^2+^ and a Cl^-^ signal in the same areas of the antennal surface, indicating a concomitant Ca^2+^ and Cl^-^ influx in OSNs. Other odorants, e.g., benzaldehyde, induced spatially non-overlapping Cl^-^ and Ca^2+^ signals, indicating independent excitation and inhibition events in distinct sensilla (Fig. 2*D*). This separation of inhibition and excitation is underlined by the strong Cl^-^ increase induced by acetic acid in the tip region of the antenna without significant Ca^2+^ responses (Fig. 2*E*). Acetic acid activates solely OSNs present in one type of coeloconic sensilla (Abuin et al., 2011), which is not labeled by the *Orco-Gal4* line. To verify that the observed antennal Cl^-^ signals indeed reflect neuronal inhibition, we expressed Clomeleon selectively in OSNs expressing Or47b. OSNs expressing this receptor selectively respond to the pheromone methyl laurate and are mainly inhibited by other odors (Hallem and Carlson, 2006; Dweck et al., 2015). Application of the odor ethyl-3-hydroxybutyrate, which induces an inhibition of Or47b-expressing OSNs as shown via single-sensillum recordings (Hallem and Carlson, 2006), leads to a strong and long-lasting Cl^-^ influx in this OSN type (Fig. 2*F*).

We next wondered whether the odor-induced antennal Cl^-^ increase derives within the sensillum and can be attributed to OSN dendrites or whether these signals rather reflect a feedback inhibition from the AL. We therefore monitored Cl^-^ signals following odor stimulation while we abolished any feedback signaling from the AL by cutting the antennal nerve (Fig. 2*G*). Interestingly, this treatment significantly reduced Cl^-^ signals in the antenna, but did not abolish them. This result demonstrates Cl^-^ conductivity in dendrites of OSNs, indicating that Cl^-^ channels are present in OSNs and localized to the most peripheral dendritic compartments in the fly antenna. However, at the same time we do not exclude an additional feedback inhibition from the AL.

### Cl^-^-dependent, inhibitory odor maps in OSN terminals in the AL

**Figure 3.**
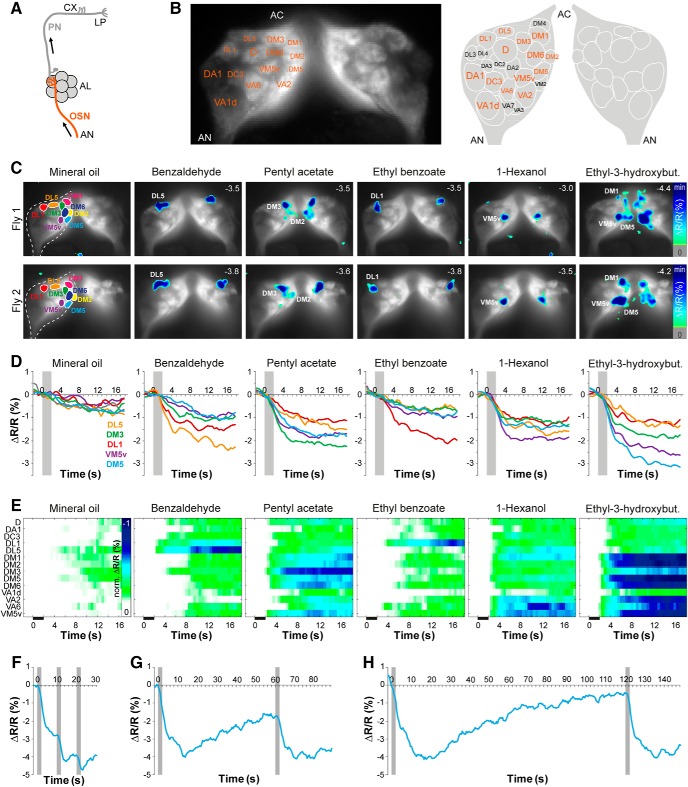
An odor-specific spatial map of chloride responses in AL sensory neurons. ***A***, Schematic illustrating the expression site of Clomeleon (AL, antennal lobe; AN, antennal nerve; CX, calyx; LP, lateral protocerebrum). ***B***, left, Clomeleon YFP baseline fluorescence in axon termini of OSNs in the AL with anatomic identification of individual glomeruli. Right, Schematic AL map viewed from the angle used for imaging experiments. Glomeruli marked in orange could reliably be identified. AC, antennal commissure. ***C***, Pseudocolor rendering of Cl^-^ responses to different odors and mineral oil in OSN axon termini in the AL of two different individuals. Images represent ΔR/R (%) superimposed onto raw fluorescence images according to the scales on the right. Numbers in each image represent individual fluorescence minimum. Glomerular positions are shown in the first image; glomeruli revealing highest Cl^-^ increase are indicated in each image. The minimum of the scaling is given in each frame in the upper right corner. ***D***, Time courses of Cl^-^ influx for each odor and mineral oil averaged across six to nine animals. Individual glomeruli are indicated by different colors, odor stimulation is marked in gray. ***E***, False color pictures of averaged odor-evoked Cl^-^ signals for 14 glomeruli (42% of all glomeruli labeled by *Orco-Gal4*) over time across six to nine animals. Clomeleon responses were normalized to highest Cl^-^ influx in each animal over all odors before averaging. Black bar indicates odor application. ***F–H***, Representative time courses of Cl^-^ influx to repeated stimulations of ethyl-3-hydroxybutyrate using interstimulus intervals of 10 s (***F***), 60 s (***G***), and 120 s (***H***). Odor stimulations are marked in gray.

Within the AL, OSNs are presynaptically inhibited by GABAergic LNs (Olsen and Wilson, 2008; Root et al., 2008; Mohamed et al., 2019) with varying and glomerulus-specific GABA sensitivities (Hong and Wilson, 2015). To visualize odor-evoked inhibition at the level of the axonal termini across multiple glomeruli, we performed Cl^-^ imaging in presynaptic OSN axons in the AL using an *in vivo* preparation (Strutz et al., 2012; Fig. 3*A*). Due to the stereotypy of the glomerular AL morphology, we could reliably identify individual glomeruli in each animal using digital AL atlases (Laissue et al., 1999; Grabe et al., 2015; Fig. 3*B*). Each odor stimulation induced a specific combinatorial pattern of inhibited glomeruli, which was stereotypic among individuals (Figs. 3*C*). The time courses of YFP/CFP ratio changes in selected glomeruli revealed an odor- and glomerulus-specific Cl^-^ influx (Fig. 3*D*). However, a time-resolved analysis across multiple glomeruli showed that Cl^-^ signals are detected in all glomeruli optically accessible during the imaging experiments (Fig. 3*E*). In conclusion, strong and odor-specific inhibition of distinct glomeruli is accompanied by less intense, global inhibition across the entire OSN population. The Cl^-^ signals that were optically monitored lasted until the end of each measurement, i.e., they strongly outlasted the 2-s odor stimulation. Therefore, we examined how much time was required before the Clomeleon signal returned to baseline (Fig. 3*F–H*). Odor application with different inter-stimulus intervals revealed that although the fluorescence emission (ΔR/R) continued to drop after stimulation, repetitive odor stimulation still elicited further Clomeleon signals after 10 or 60 s (Fig. 3*F*,*G*). A complete recovery of the Clomeleon fluorescence was not observed before 120 s after odor stimulation had elapsed (Fig. 3*H*). The actual kinetics of any fluorescence sensor depend on multiple factors, e.g., the concentration of the sensor determined by the expression level, the affinity of the sensor to its ligand, or the dynamic range of the sensor. Therefore, it is difficult to conclude to what degree the dynamics of Cl^-^ transients quantitatively reflect the actual balance between Cl^-^ influx and intracellular Cl^-^ removal. However, a slow recovery of Clomeleon signals has also been observed in mammalian neurons (Kuner and Augustine, 2000; Berglund et al., 2006) and has been attributed to the slow removal of [Cl^-^]_i_ by transporters rather than kinetic properties of the Cl^-^ sensor (Staley and Proctor, 1999; Berglund et al., 2009, 2016). It is therefore quite conceivable that the odor-evoked Cl- transients in OSNs indeed strongly outlast the actual stimulation.

### Comparison between odor-evoked Cl^-^ signaling in OSN dendrites and axons

**Figure 4.**
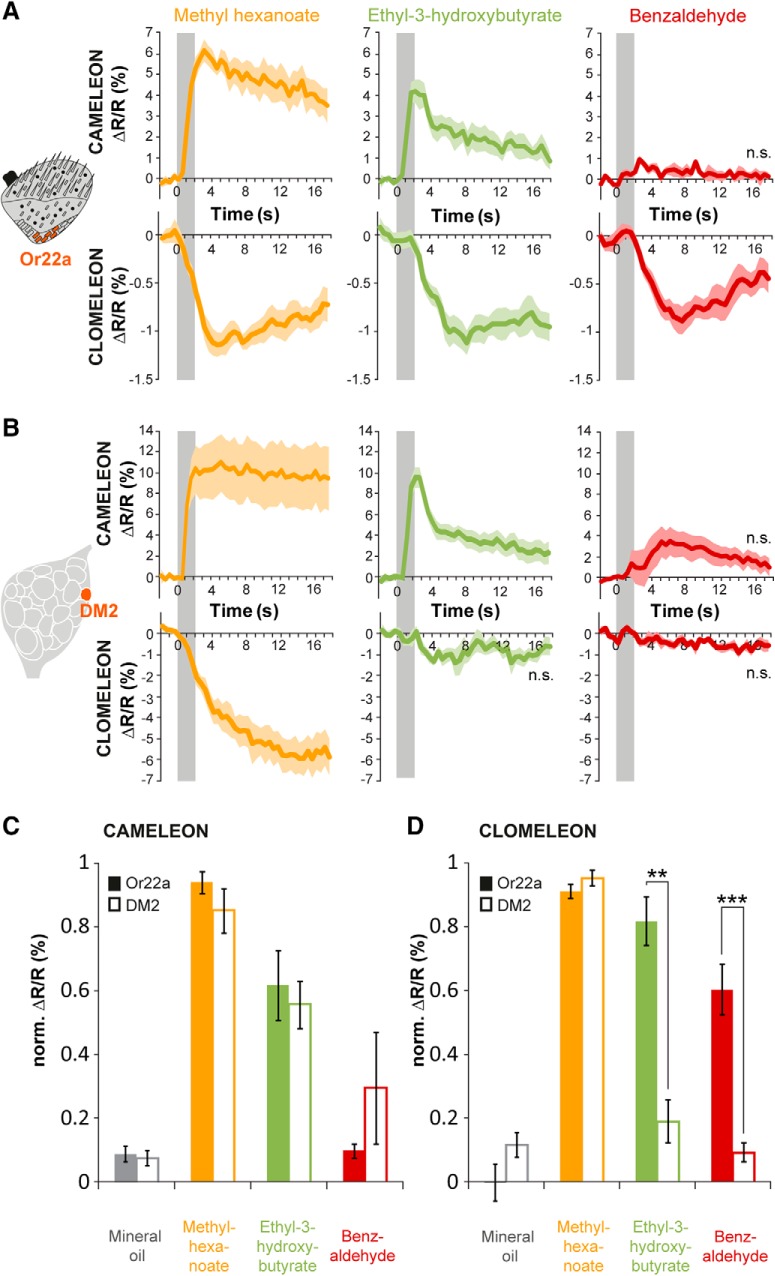
Chloride responses are modulated on their way from the antenna to the AL. ***A***, left, Schematic of the third antennal segment illustrating selective expression of Cameleon or Clomeleon in dendrites and somata of Or22a-expressing OSNs. Right, Averaged time courses of Ca^2+^ (upper row) and Cl^-^ influx (lower row) in Or22a-expressing OSNs in the fly antenna to three different odors. Odor stimulation is indicated in gray. Lines represent means, color shadings represent SEM (n.s. not significant from solvent; *n* = 6–7). ***B***, left, Schematic of the *Drosophila* AL indicating selective expression of Cameleon or Clomeleon in axonal termini of Or22a-expressing OSNs which converge to glomerulus DM2. Right, Averaged time courses of Ca^2+^ (upper row) and Cl^-^ influx (lower row) in DM2 to three different odors. Odor stimulation is marked in gray. Lines represent means, color shading represents SEM (*n* = 6). ***C***, ***D***, Quantification of Ca^2+^ (***C***) and Cl^-^ (***D***) influx in Or22a-expressing OSNs to three different odors and mineral oil. Data are shown as pair-wise comparisons between antenna (Or22a) and AL (DM2). Clomeleon and Cameleon responses have been normalized to highest Cl^-^ or Ca^2+^ influx in each animal over all odors, respectively. Cl^-^ responses to ethyl-3-hydoxybutyrate and benzaldehyde are significantly lower in the AL compared to the antenna (**p < 0.01, ***p < 0.001, Mann–Whitney test, *n* = 6–7).

As shown so far, odors induce a clear Cl^-^ increase at the level of the peripheral signal input, i.e., in the antenna (Fig. 2), and at the sites of synaptic transmission, i.e., in OSNs of the AL (Fig. 3). To examine the relationship between these two signal sources in more detail, we comparatively monitored odor-evoked [Cl^-^]_i_ and [Ca^2+^]_i_ of a single OSN population at its dendrites and axonal termini. This was achieved by selective expression of Clomeleon or Cameleon, respectively, in OSNs expressing Or22a, which targets the glomerulus DM2 (Couto et al., 2005; Fishilevich and Vosshall, 2005). As described previously (Pelz et al., 2006), a strong Ca^2+^ response was elicited by methyl hexanoate, while ethyl-3-hydroxybutyrate induced an intermediate, and benzaldehyde no significant response (Fig. 4*A*,*B*). The relative intensities of odor-evoked Ca^2+^ responses did not differ between antenna and AL (Fig. 4*C*). However, all three odors induced comparatively strong Cl^-^ responses in the fly antenna (Fig. 4*A*, lower panel), while only methyl hexanoate, one of the most potent activators of this OSN type, elicited a significant Cl^-^ response at the AL level (Fig. 4*B*, lower panel, *D*). Hence, the intensity of odor-evoked Cl^-^ influx at the level of OSN dendrites and somata is relatively independent of the actual intensity of the accompanying Ca^2+^ influx. On the contrary Cl^-^-mediated inhibition in the AL reflects more odor-specific inhibition.

### Cl^-^-dependent, inhibitory odor maps in PN dendrites in the AL

**Figure 5.**
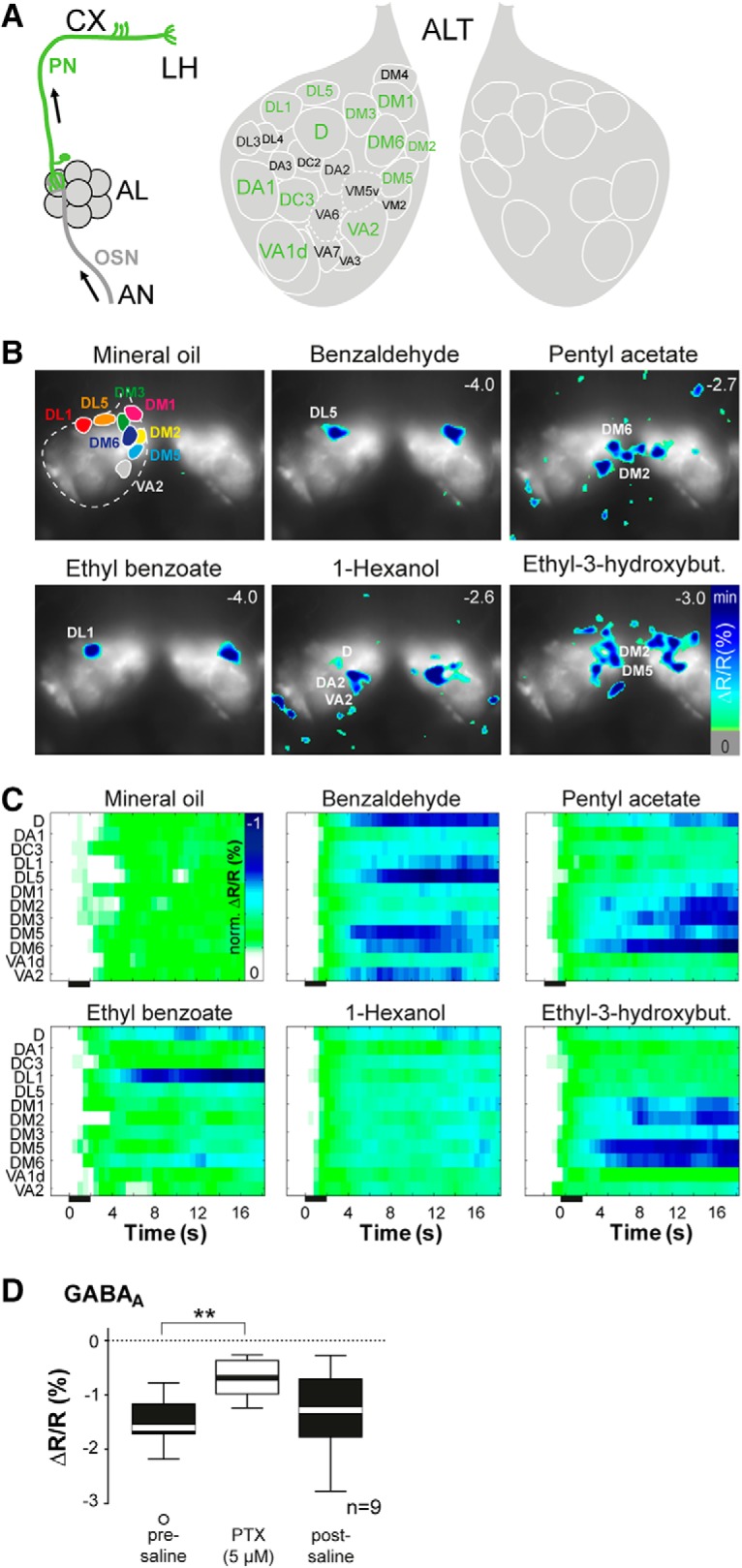
GABA_A_ receptors contribute to odor-evoked chloride responses in PNs. ***A***, left, Schematic illustrating expression site of Clomeleon. Middle, AL map viewed from the angle that was used for imaging. Glomeruli indicated in green could reliably be identified. Right, Contralateral AL including reliably identified glomeruli. ALT, AL tract. ***B***, Pseudocolor rendering of representative Cl^-^ responses to different odors and mineral oil in PN dendrites in the AL. Images represent ΔR/R (%) superimposed onto raw fluorescence images according to the scale on the right. Numbers in each image give the individual fluorescence minimum. Glomerular positions are shown in the first image; individual glomeruli revealing highest Cl^-^ increase are indicated in each image. ***C***, False color pictures of averaged odor-evoked Cl^-^ signals for 12 identified glomeruli (40% of all glomeruli labeled by *GH146-Gal4*) over time across 9–11 animals. Clomeleon responses were normalized to highest Cl^-^ influx in each animal over all odors before averaging. Black bar indicates the odor application. ***D***, Quantification of Cl^-^ influx to ethyl-3-hydroxybutyrate in PNs before, during and after applying of picrotoxin. The GABA_A_ receptor blocker significantly reduces odor-evoked Cl^-^ responses (**p < 0.01, repeated measures ANOVA, *n* = 9).

To analyze inhibitory patterns of output neurons in the AL, we performed Cl^-^ imaging at the dendrites of PNs using the enhancer trap line *GH146-Gal4* that labels the majority of uniglomerular PNs (Stocker et al., 1997). Odor application induced clear spatially confined and odorant-specific patterns of inhibition that could be assigned to identified glomeruli (Fig. 5*A*,*B*). A time-resolved analysis across all glomeruli revealed a strongly pronounced Cl^-^ influx in a glomerulus- and odor-specific manner, and typically with some delay after odor onset (Fig. 5*C*). These odor-specific, inhibitory patterns evolve slowly over time and persist until the end of the measurement, as it is the case at the OSN level. Notably, we observed a concordance in the Cl^-^ responses between OSNs and PNs, in a way that a given odor inhibited the same glomeruli at the input and the output level of the AL (Figs. 3*E*, 5*C*). However, this correlation was only apparent for strongly inhibited glomeruli, while weaker Cl^-^ responses occurred in more glomeruli at the PN level when compared to OSNs. Again, this indicates a dual role of Cl^-^-mediated inhibition, i.e., a moderate, global inhibition and a strong odor- and glomerulus-specific inhibition, potentially reflecting the various types of inhibitory neurons in the AL, the global and patchy GABAergic LNs (Chou et al., 2010; Mohamed et al., 2019).

Since the spatiotemporal activity of PN ensembles is influenced by inhibitory, GABAergic LNs (Wilson and Laurent, 2005), we tested whether the Clomeleon signals were dependent on GABA receptors. Therefore, we performed Cl^-^ imaging experiments after silencing the inhibitory LN input by applying the GABA_A_-type antagonist picrotoxin (5 μM) that blocks ionotropic Cl^-^-ion channels. In addition to GABA_A_ receptors, picrotoxin has been shown to block also glutamate-gated chloride channels (GluCl; Liu and Wilson, 2013). However, at the low concentration used in this study the antagonist mainly functions as a GABA_A_ antagonist without affecting GluCl channels (Hong and Wilson, 2015). Application of picrotoxin led to a significant reduction of the odor-induced Cl^-^ signals by on average 59% (Fig. 5*D*). This result indicates that the GABA_A_ receptor contributes to the Cl^-^-mediated inhibition at the AL output level.

### A comparative functional map of odor-evoked activation and inhibition in the AL

**Figure 6.**
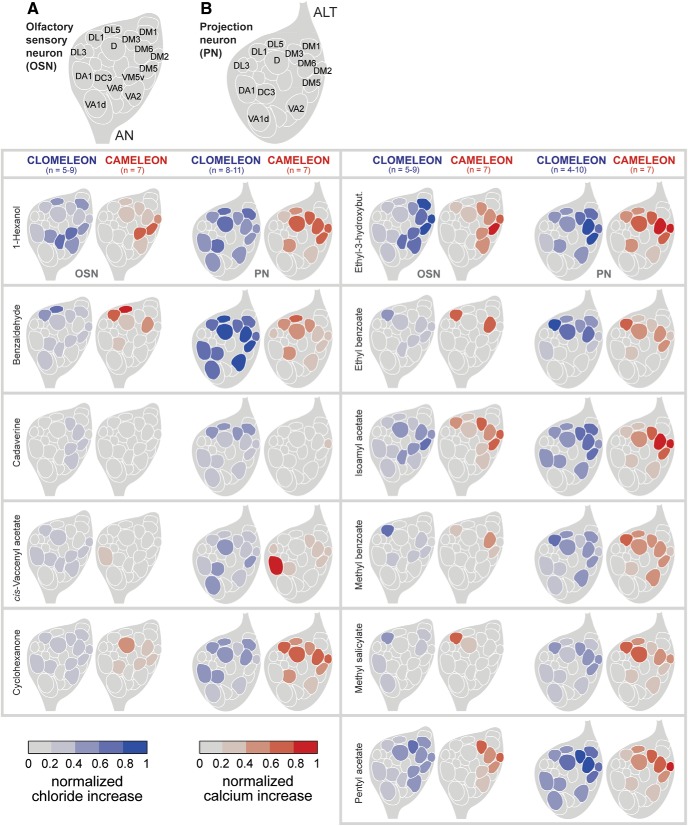
A functional map of odor-evoked inhibition and excitation. ***A***, ***B***, Averaged odor-evoked Cl^-^ (left, in blue) and Ca^2+^ (right, in red) responses in OSNs (***A***) and PNs (***B***) are represented as schematic ALs for 11 odors according to the scales below. Responses were normalized to highest Cl^-^ or Ca^2+^ influx in each animal over all odors. Glomerular identities are indicated by AL maps at the top. AC, antennal commissure; AN, antennal nerve; ALT, AL tract.

We next examined the overlap of the odor-evoked inhibitory patterns compared to the spatial patterns of glomerular Ca^2+^ activities. To do so, we performed functional imaging experiments to a variety of different odors and monitored odor-evoked Ca^2+^ as well as Cl^-^ responses by expressing Cameleon or Clomeleon in OSNs and PNs, respectively. Subsequently, we mapped the odor-induced responses to identified glomeruli to generate a functional AL atlas (Fig. 6). First, we observed that, in the majority of cases, the odor-evoked maps of excitation and inhibition closely match at the input and the output level, i.e., those glomeruli which were excited were also often inhibited by a certain odor. Such a concordance suggests a gain control mechanism for odor-induced excitation as described for the OSN level (Olsen and Wilson, 2008), which should occur in all glomeruli receiving an excitatory input. Second, we observed that some glomeruli were inhibited without being excited. This finding suggests a second role of Cl^-^-mediated inhibition in the *Drosophila* AL which could contribute to confining the spatiotemporal patterns, resulting in an enhanced contrast between different odor representations as shown for the honeybee AL (Sachse and Galizia, 2002). Notably, we never observed glomeruli, which were excited without being inhibited.

### Input-output transformation

**Figure 7.**
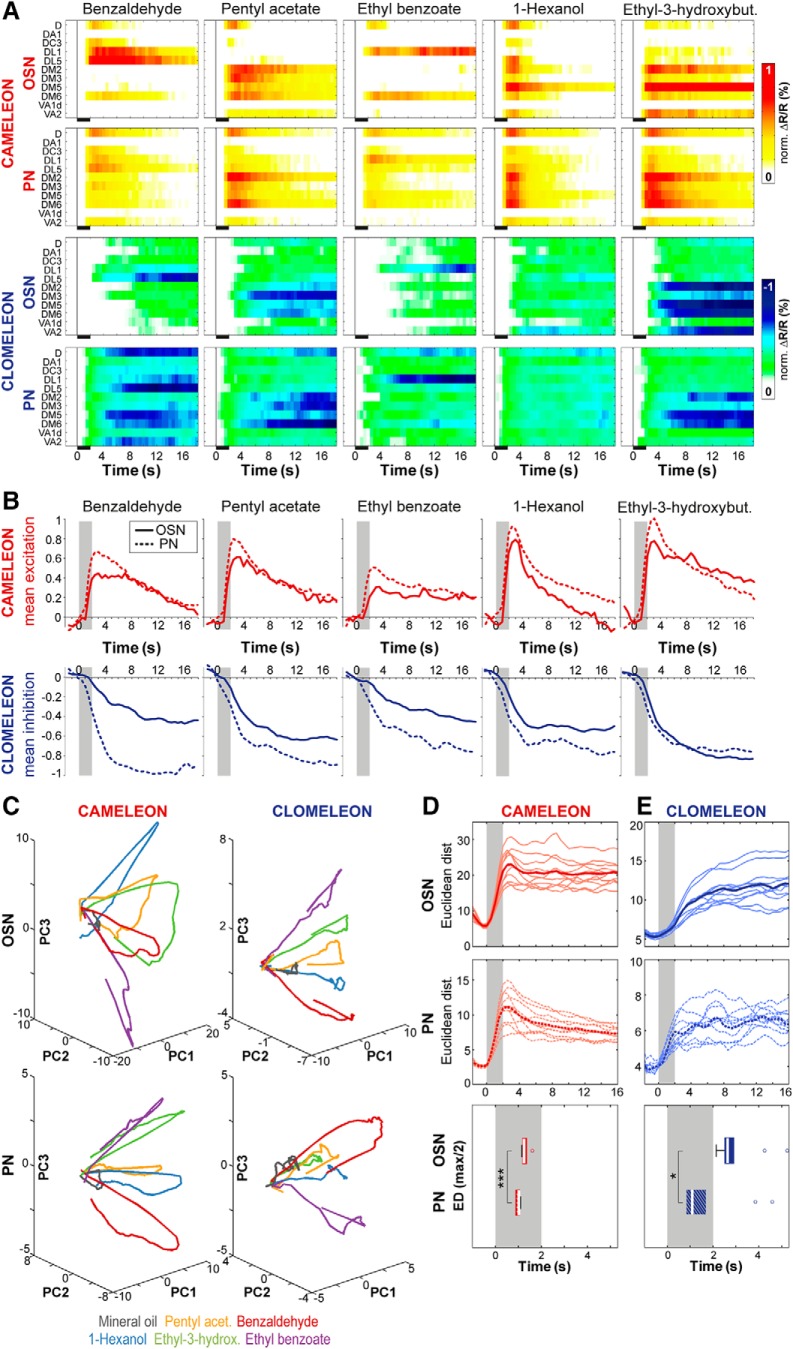
Input-output transformation of odor-evoked Ca^2+^ and Cl^-^ responses. ***A***, False colored activity of averaged odor-evoked Ca^2+^ (white-yellow-red) and Cl^-^ (white-green-blue) influx to different odors for the same set of glomeruli in OSNs (upper panels) and PNs (lower panels) over time. Responses were normalized to highest Cl^-^ or Ca^2+^ influx in each animal over all odors before averaging. Black bars indicate odor application. ***B***, Time courses of mean excitation (above, red) and inhibition (below, blue) to different odors averaged over all glomeruli and animals for OSNs (solid line) and PNs (dotted line). Odor stimulation is given by a gray bar. Cameleon, *n* = 7; Clomeleon, *n* = 9–11. ***C***, Odor separation visualized using principal component analysis. The first three principal components account for 67.3% (OSNs: Cameleon), 67.4% (OSNs: Clomeleon), 79.7% (PNs: Cameleon), and 59% (PNs: Clomeleon) of the variation in the related data set. Plotting the first three principal components reveals odor-specific trajectories of ensemble activity in OSNs (upper panels) and PNs (lower panels). ***D***, upper two panels, Time-resolved Euclidean distances (ED) between population vectors of different odor representations using Cameleon. Odor stimulation is marked in gray. Distances were calculated separately for OSN (solid lines) and PN (dotted lines) responses. Individual pair-wise odor distances are given by thin lines, averaged Euclidean distances are shown in bold. Lower panel, Latency to half maximal odor separation based on normalized Euclidean distances for 10 pair-wise odor combinations (individual lines in ***B***) for Ca^2+^ signals in OSNs and PNs. PNs reach half maximum odor separation significantly earlier than OSNs (***p < 0.001, two-tailed paired *t* test; *n* = 7). ***E***, Same as in ***D*** for Clomeleon-derived odor responses. Half maximum odor separation based on odor-evoked Cl^-^ responses occurs significantly earlier in PNs than in OSNs (*p < 0.05, two-tailed paired *t* test; *n* = 9–11).

Last, we analyzed the difference between the odor-evoked representations of input and output neurons for a subgroup of 11 glomeruli that could be unambiguously identified in each experiment (Fig. 7*A*). Since each fluorescent reporter protein exhibits different kinetics, one has to be careful when comparing temporal dynamics between different sensors. We therefore compared temporal aspects of odor-evoked responses of different processing levels for one reporter protein only. Quantification of the evoked mean responses to specific odors showed that excitatory as well as inhibitory odor responses were, on average, stronger at the PN level than at the OSN level (Fig. 7*B*) which is well in line with electrophysiological recordings (Wilson and Laurent, 2005; Bhandawat et al., 2007; Seki et al., 2017). To visualize how the odor-specific responses evolve over time, we applied principal component analyses to reduce the multidimensional, spatiotemporal activity/inhibition to three dimensions and illustrated the odor-evoked ensemble activity as trajectories over time (Fig. 7*C*). Independent of the reporter protein, different odors evoked distinct trajectories, which demonstrate an odor-specific separation of Ca^2+^ as well as Cl^-^ responses at both processing levels, i.e., OSNs and PNs. To quantify how fast this odor separation evolved, we calculated Euclidean distances between the population vectors of the different odor representations for Cameleon and Clomeleon signals, respectively (Fig. 7*D*,*E*, upper panels). Interestingly, PN responses revealed in general lower Euclidean distances than OSN responses. Although PNs showed an increased level of inhibition, they also exhibited generally broader odor-evoked responses compared to OSNs (Wilson et al., 2004; Seki et al., 2017). This broadening leads to wider odor tuning curves and a stronger overlap of odor representations at the PN level (Niewalda et al., 2011; Schubert et al., 2014; Seki et al., 2017), while PN responses show a higher degree of odor categorization according to behaviorally meaningful values (Niewalda et al., 2011; Knaden et al., 2012).

After normalizing all pair-wise Euclidean distances, we calculated the latencies to the half maximum odor separation (Fig. 7*D*,*E*, lower panel) and observed that it was reached significantly earlier in PNs than in OSNs. This finding is in accordance with electrophysiological recordings in *Drosophila* showing that PN responses have shorter latencies to reach 90% of their response peak than OSNs (Bhandawat et al., 2007) indicating that PNs act as high-pass filters that rapidly convey rising OSN responses to third-order neurons. When considering the Clomeleon responses, this latency shift is even more pronounced for Cl^-^ signals. This observation is most likely due to reciprocal inhibitory mechanisms that differently affect OSN and PN responses: PNs are inhibited by fast forward inhibition from OSNs via GABAergic LNs (Wilson and Laurent, 2005) before OSNs receive presynaptic feedback inhibition from PNs through, in turn, GABAergic LNs (Olsen and Wilson, 2008; Root et al., 2008).

## Discussion

### Clomeleon-based Cl^-^ imaging in the *Drosophila* nervous system

Hardly any optical imaging technique reaches the unmatched temporal precision of electrophysiological recordings as yet, and the determination of membrane potential changes represents the most accurate approach to determine how sensory stimuli are represented by single or small groups of neurons (Wilson et al., 2004; Wilson and Laurent, 2005; Seki et al., 2017). Optical imaging, on the contrary, offers the advantage of monitoring physiologic parameters that correlate with membrane potential changes across spatiotemporally distributed populations of neurons (Ahrens et al., 2013; Chen et al., 2013). Membrane depolarization is typically accompanied by increases in intracellular Ca^2+^ from a variety of sources, and Ca^2+^ imaging represents currently the “gold standard” for visualizing neuronal excitation in *Drosophila* (Riemensperger et al., 2012). However, neuronal inhibition, most often mediated by Cl^-^ influx, is not directly captured using Ca^2+^ imaging. Establishing Clomeleon as a tool for monitoring Cl^-^ dynamics both in the peripheral and central nervous system provides an important step toward filling this gap. Its ratiometric nature as a FRET-based sensor demands some additional considerations in contrast to single chromophore sensors such as calcium reporters belonging to the GCaMP family (Tian et al., 2009). Especially the size, pH sensitivity, and slow response dynamics require the future development of a single chromophore chloride sensor, which hopefully eases its applicability. The development of the Cl^-^ sensor SuperClomeleon, which still represents a FRET-based sensor, reveals an improved signal-to-noise ratio and needs to be established for the *Drosophila* olfactory system (Grimley et al., 2013).

It is important to consider that the monitored changes in intracellular Ca^2+^ and Cl^-^ derive from different cell processes within the neurons. Recorded changes in Ca^2+^ and Cl^-^ can therefore depend on fluxes at the synapse or along the neuron, as well as release from intracellular calcium stores, which are mediated by ligand-gated as well as voltage-gated Ca^2+^ and Cl^-^ channels in insects (Messina et al., 1996; Wicher et al., 2001; Fiala and Spall, 2003; Flores et al., 2006; Pézier et al., 2010). Since the temporal resolution in functional imaging recordings is rather low compared to electrophysiological recordings, the different dynamics of these ion channels are not visible in the fluorescence signal of the different sensors.

### Is Cl^-^ influx part of the olfactory signal transduction in insects?

We observed an odor-evoked Cl^-^ influx in OSN dendrites of the *Drosophila* antenna. In vertebrates, Cl^-^-conductance is an integral component of the canonical olfactory signal transduction cascade (Labarrera et al., 2013). Here, odor stimulation leads to a membrane-current composed of a cationic and a delayed Cl^-^ component (Kurahashi and Yau, 1993). Although Cl^-^-conductance is in most cases associated with neuronal inhibition, this Cl^-^ current amplifies the olfactory signal by Cl^-^ efflux through a Ca^2+^-activated Cl^-^ channel which is most likely mediated by anoctamin-2 (ANO2; Lowe and Gold, 1993; Stephan et al., 2009; Delgado et al., 2016). The insect olfactory signal transduction is crucially different from that of vertebrates in two aspects: First, olfactory receptors of the OR and IR type are ionotropic receptors mediating excitatory cation influx (Sato et al., 2008; Wicher et al., 2008; Rytz et al., 2013). Metabotropic signaling cascades have been clearly described for insect OSNs, but their exact modulatory functions remain unclear as yet (Wicher et al., 2008). Second, the equilibrium potential of Cl^-^ (*E_Cl_*) in insect OSNs differs from that of vertebrates. Since [Cl^-^]_i_ is lower than in the extracellular medium, as shown in moths (Steinbrecht, 1992), the electromotive force will lead to a Cl^-^ influx, if the membrane potential is shifted above *E_Cl_* (i.e., –36 mV). Hence, when OSNs become excited, a Cl^-^ influx through Ca^2+^-activated Cl^-^ channels might result in hyperpolarization of the plasma membrane (Pézier et al., 2010).

Interestingly, dendrites of moth OSNs express an analogous Ca^2+^-activated Cl^-^ channel that functionally resembles ANO2 (Pézier et al., 2010). The *Drosophila melanogaster* genome contains two different ANO2 orthologues (CG6938, CG10353) whose molecular function has, however, not yet been studied. Additional experiments are needed to analyze the role of ANO2 in odor-evoked Cl^-^ dynamics in *Drosophila* OSNs. The fact that the antennal Cl^-^ influx co-occurred frequently with a Ca^2+^ influx further suggest the existence of Ca^2+^-activated Cl^-^ channels in the antenna. This type of Cl^-^-mediated inhibition might reflect shunting inhibition as a mechanism for gain control leading to stabilization of odor-evoked excitation (Wilson and Mainen, 2006).

In addition, we also observed Cl^-^ influx that was not directly correlated with the excitation of the respective OSNs, reflecting a second type of Cl^-^-mediated inhibition. This finding suggests either again the existence of Cl^-^-channels in OSN dendrites or, alternatively, a retrograde diffusion of Cl^-^ from the AL. Interestingly, when we abolished any feedback signaling from the AL, we still observed Cl^-^ influx, supporting the first assumption. However, since the Cl^-^ signals were not identical but reduced, we assume that Cl^-^ dynamics in OSN dendrites are partly influenced by Cl^-^ influx into OSN axonal termini in the AL. The latter assumption is further supported by our observation that applying GABA to the AL induced a significant Cl^-^ influx in the antenna (data not shown).

The comprehensive study by Hallem and Carlson on receptor-ligand interactions where a widespread inhibition below baseline firing rates among one third out of 24 selectively expressed ORs was observed (Hallem and Carlson, 2006), is well in line with our observation of inhibitory odor responses in the *Drosophila* antenna. Interestingly, OSNs expressing Or47b, known to selectively respond to the pheromone methyl laurate (Dweck et al., 2015), were never excited by the large odor set tested in the aforementioned study, but showed inhibitory responses to 34% of the odors. Those OSNs target glomerulus VA1d, and we indeed observed clear odor-evoked Cl^-^ responses in VA1d, while Ca^2+^-influx did never occur. In addition, Cl^-^ imaging of Or47b-expressing OSNs on the antenna confirms the odor-induced inhibition of this OSN type. As a second example, benzaldehyde elicited a strong Cl^-^ influx in OSNs expressing Or22a in the antenna without being accompanied by a Ca^2+^ influx. This odor has already been characterized as an Or22a-inhibitor (Pelz et al., 2006; Wicher et al., 2008), which strongly suggests that our second type of Cl^-^-mediated inhibition reflects hyperpolarization and thus odor-specific inhibition in the antenna. Therefore, our study demonstrates that inhibitory odor responses of OSNs are not only generated by a reduction in the intracellular cation concentration leading to a reduced firing rate as widely assumed, but that they are also carried by an influx of Cl^-^. It still remains to be investigated how Cl^-^ channels are integrated in the olfactory signal transduction machinery of insects.

### Multiple roles of Cl^-^ signaling at the AL network level

Within the insect AL, odor representations are shaped by the inhibitory network of various types of GABAergic LNs (Sachse and Galizia, 2002; Wilson and Laurent, 2005; Silbering and Galizia, 2007; Hong and Wilson, 2015; Mohamed et al., 2019). It has been shown that OSNs are presynaptically inhibited by LNs, mediated by both GABA_A_ and GABA_B_ receptors (Olsen and Wilson, 2008; Root et al., 2008). Since GABA_A_ receptors are ligand-activated Cl^-^ channels, they provide a direct molecular substrate for the Cl^-^ influx in OSNs at the AL level. Likewise, PNs express both GABA_A_ and GABA_B_ receptors (Enell et al., 2007), and their odor responses are influenced by both receptor types (Wilson and Laurent, 2005; Silbering and Galizia, 2007). Here we confirm the contribution of GABA_A_ receptors pharmacologically for Cl^-^ influx. In addition, our data provide evidence that the synaptic inhibition of PNs is stronger than that of OSNs, since we clearly see an increase in the number of inhibited glomeruli from the input to the output level. However, one has to keep in mind that the sensor dynamics might not reflect the potentially varying dynamics of the membrane potential in these different neuron types. Chloride ions themselves have their own dynamics, and potentially those dynamics reflect actual neuronal dynamics only loosely. Still, our data demonstrates a transformation of odor representations that is not accessible if only excitation-associated Ca^2+^ is taken into account. Our findings suggest two distinct types of Cl^-^ signals in the AL, i.e., a global, moderate inhibition and a strong, cell-type-specific inhibition. This reflects the structural diversity of GABAergic LNs in the *Drosophila* AL (Chou et al., 2010; Seki et al., 2010; Hong and Wilson, 2015). The majority of LNs arborizes in most glomeruli, and therefore evenly distributes the input from most OSN types. Thus, we would expect that the level of inhibition in each glomerulus should mirror the level of activity in all glomeruli with varying sensitivities to the GABAergic input (Hong and Wilson, 2015). This assumption provides a mechanism for global, inhibitory gain control at the cellular and network level to keep the olfactory circuitry in the operating state across odorant combinations and concentrations as shown for the zebrafish olfactory bulb (Zhu et al., 2013).

As a second type of Cl^-^-mediated inhibition, we observed Cl^-^ responses that were not linked to any excitation, and most likely reflect local inhibition that specifically shapes neuronal information processing, analogous to the mammalian system (Mori et al., 1999; Urban, 2002). In fact, heterogeneous populations of LNs innervating only few glomeruli also exist (Chou et al., 2010; Seki et al., 2010), which might provide the neuronal substrate for such glomerulus- and odor-specific inhibition. Along that line, recent data provide evidence that patchy, but not global GABAergic LNs accomplish selective lateral inhibition between specific glomeruli processing odors with opposing hedonic valences (Mohamed et al., 2019).

### Temporal aspects of odor-evoked chloride responses

The measured odor-induced Cl^-^ and Ca^2+^ responses reveal different temporal dynamics. However, the temporal differences between Ca^2+^- and Cl^-^-evoked signals are difficult to interpret because it is not clear whether they derive from different reporter dynamics or indeed reflect physiologic properties. Hence when considering temporal dynamics, we restricted any comparison of data obtained to only one reporter protein and therefore compared dynamics of input and output neurons for Cameleon and Clomeleon separately.

Although the chloride influx is clearly odor-induced, it evolves slowly over time and outlasts the odor stimulation period. Such long-lasting chloride responses are consistent with observations in mammalian neurons (Kuner and Augustine, 2000; Berglund et al., 2006) and might reflect the relatively slow rate of Cl^-^ removal from the neurons (Staley and Proctor, 1999; Berglund et al., 2009, 2016). This slow recovery in the Cl^-^ response might affect the excitability of the neuron for a period significantly outlasting the stimulation. However, as mentioned above, the kinetics of fluorescence sensors depend on intrinsic parameters of the sensor itself and firm conclusions about the exact kinetics about the Cl^-^ currents cannot be drawn as yet.

### Determining Cl^-^ and Ca^2+^ representations together provide a more accurate assessment of sensory processing

The importance of synaptic inhibition for accurate behavioral responses to olfactory stimuli has been demonstrated in different species. Mice show accelerated discrimination ability when synaptic inhibition of mitral cells is increased by selectively altering granule cell function (Abraham et al., 2010). In locusts and flies, disruptive manipulation of the GABAergic AL network reduces the insects’ ability to behaviorally discriminate between similar odors (Stopfer et al., 1997; Barth et al., 2014). The similarity between glomerular excitation patterns evoked by different odors often matches with the animals’ ability to discriminate between the odors in behavioral tasks (Sachse and Galizia, 2003; Guerrieri et al., 2005; Niewalda et al., 2011; Barth et al., 2014; Carcaud et al., 2018). In *Drosophila*, the spatiotemporal, glomerular Ca^2+^ activity patterns at the PN level reflect more accurately the animals’ perception of similarities between odors than the patterns observed at the OSN level (Niewalda et al., 2011). This difference between OSNs and PNs could, at least partly, be due to the influence of GABA-mediated inhibition. Determining Cl^-^-mediated inhibition across ensembles of neurons in addition to Ca^2+^-mediated excitation therefore enables us to more comprehensively and more accurately characterize sensory processing underlying the perception of olfactory or other sensory stimuli.

## Synthesis

Reviewing Editor: Anna Menini, SISSA (International School of Advances Studies) Decisions are customarily a result of the Reviewing Editor and the peer reviewers coming together and discussing their recommendations until a consensus is reached. When revisions are invited, a fact-based synthesis statement explaining their decision and outlining what is needed to prepare a revision will be listed below.

This study addresses an interesting and important topic in sensory physiology, as not only seeks new insights into the function of the insect olfactory system, but also provides important technical insights into the use of genetically encoded C- indicators.

The Reviewers have specific suggestions for improving the manuscript related to data analysis and interpretation. Please carefully address the specific concerns listed below.

Major comments

The methods are described carefully and in detail and the quality of the data is very good. However, given the experimental design, the interpretation of some aspects of data should be revised. The authors should take a more critical look at their data and try to identify parameters in the data that are caused (or can be caused) by the experimental approach and verify whether conclusions can really be justified by data.

1) It is important to state and consider VERY clearly what the principal differences between the measured Cl- signal and the Ca2+ signal are. Cl- flux through the membrane is almost exclusively mediated by ligand gated channels (mainly at the synapse). In contrast Ca2+ flux through the membrane is mediated by ligand gated channels (mainly at the synapse) AND voltage ('activity') gated channels (all over the neurons). Obviously this has to be taken into account when constructing (spatial) 'functional excitation/inhibition maps'.

2) Another important point to consider for the interpretation of the data is the experimental design including the stimulation protocol, frame rate and properties/concentration of the ion indicators (which can significantly influences the kinetics of the signals). The stimulation lasted 2s, the recording time was ~20s and the frame rate was 2Hz. This allows only a very limited temporal resolution during the stimulus (?!). Here the analysis focused on the signal several seconds AFTER the stimulation. How physiologically relevant is that? This question is particularly relevant since we know, at least for Ca2+, that signal amplitude and kinetics are significantly dependent from non-physiological parameters including indicator concentration and dissociation constant. For the Cl- indicator we do not know even that. Therefore it is unclear what could be the physiological relevance of analyzing and comparing Cl- and Ca2+ kinetics under the given experimental conditions (including Fig. 7).

Minor comments

1) line 165: Glomerulus “VM5” is traditionally divided into two glomeruli (VM5v and VM5v). This should be spelled out to avoid confusion. In addition, the authors describe VA6 as not labeled by GH146-Gal4, which is incorrect (Grabe et al, 2015, J Comp Neurol).

2) Line 203-204, and Figure 1: The biphasic responses of Clomeleon to GABA application should be interpreted in more detail. It would be interesting to have the authors' perspective on what causes this dynamics response to GABA.

3) Lines 383-384 and Figure 7C,D: The authors should explain why (for both Cameleon and Clomeleon) the Euclidean distances are larger in ORNs than in PNs. Olsen et al (2010, Neuron) have reported that inhibition in the antennal lobe increases the discriminability of odors, i.e. PNs should have larger Euclidean distances than ORNs. Methodologies are different in important ways between these two studies, but it is important for the authors to explain why this discrepancy arises, as it may inform future studies.

4) Lines 466-467: “widespread inhibition below baseline firing rates among 24...” This sentence is a bit misleading, as if the authors were suggesting that all 24 of the ORs tested by Hallem and Carlson (2006, Cell) conferred significant odor-specific suppression of firing rates upon their ORNs. A slight re-wording would help.

## Author Response

Response to reviewer comments (eN-NWR-0213-19)

SYNTHESIS OF REVIEWS

(1) REVIEWER: This study addresses an interesting and important topic in sensory physiology, as not only seeks new insights into the function of the insect olfactory system, but also provides important technical insights into the use of genetically encoded C- indicators. The Reviewers have specific suggestions for improving the manuscript related to data analysis and interpretation. Please carefully address the specific concerns listed below.

Major comments: The methods are described carefully and in detail and the quality of the data is very good. However, given the experimental design, the interpretation of some aspects of data should be revised. The authors should take a more critical look at their data and try to identify parameters in the data that are caused (or can be caused) by the experimental approach and verify whether conclusions can really be justified by data.

(1) RESPONSE: Thank you very much for positive feedback and constructive criticism! We have thoroughly revised our manuscript according to all suggestions and have tried to clarify all points of criticism.

(2) REVIEWER: 1) It is important to state and consider VERY clearly what the principal differences between the measured Cl- signal and the Ca2+ signal are. Cl- flux through the membrane is almost exclusively mediated by ligand gated channels (mainly at the synapse). In contrast Ca2+ flux through the membrane is mediated by ligand gated channels (mainly at the synapse) AND voltage ('activity') gated channels (all over the neurons). Obviously this has to be taken into account when constructing (spatial) 'functional excitation/inhibition maps'.

(2) RESPONSE: We agree with the reviewers that it is important to consider the different sources for neuronal increase in intracellular calcium and chloride. In comparison with cation channels little is known, in insects, about chloride channels other than those gated by

ligands as reviewed by Wicher et al. (Prog. Neurobiol., 2001), which comprise GABA-A, glutamate-gated and calcium-activated chloride channels, such as anoctamin and bestrophin (Chien & Hartzell, J. Gen. Physiol., 2007; Liu and Wilson, PNAS, 2013; Pézier et al., J. Neurosci., 2010). However, in Drosophila a mitochondrial voltage-gated anion- selective channel (VDAC/Porin) has been cloned and characterized (Messina et al, FEBS Lett., 1996; Ryerse et al., Biochim. Biophys. Acta, 1997) as well as a hyperpolarization

dependent voltage-gated chloride channel ((DmClC-2) Flores et al., Mol. Membr. Biol., 2006; Wang et al., Mol. Med. Rep., 2017) which are not necessarily restricted to the synapse. Hence, the calcium and chloride responses measured in this study are most likely both mediated by ionotropic as well as metabotropic channels. Since the temporal resolution in functional imaging recordings is rather low compared to electrophysiological recordings, the different dynamics of these ion channels won't be distinguishable in the fluorescence signal. In order to discuss this point, we have added the following paragraph to our discussion, mentioning the different sources for the calcium and chloride responses:

“It is important to consider that the monitored changes in intracellular Ca2+ and Cl- derive from different cell processes within the neurons. Recorded changes in Ca2+ and Cl- can therefore depend on fluxes at the synapse or along the neuron, as well as release from

intracellular calcium stores, which are mediated by ligand-gated as well as voltage-gated

Ca2+ and Cl- channels in insects (Fiala and Spall, 2003; Flores et al., 2006; Messina et al.,

1996; Pézier et al., 2010; Wicher et al., 2001). Since the temporal resolution in functional imaging recordings is rather low compared to electrophysiological recordings, the different dynamics of these ion channels are not visible in the fluorescence signal of the different sensors.” (Page 19)

(3) REVIEWER: 2) Another important point to consider for the interpretation of the data is the experimental design including the stimulation protocol, frame rate and properties/concentration of the ion indicators (which can significantly influences the kinetics of the signals). The stimulation lasted 2s, the recording time was ~20s and the frame rate was 2Hz. This allows only a very limited temporal resolution during the stimulus (?!). Here the analysis focused on the signal several seconds AFTER the stimulation. How physiologically relevant is that? This question is particularly relevant since we know, at least for Ca2+, that signal amplitude and kinetics are significantly dependent from non-

physiological parameters including indicator concentration and dissociation constant. For the

Cl- indicator we do not know even that. Therefore it is unclear what could be the physiological relevance of analyzing and comparing Cl- and Ca2+ kinetics under the given experimental conditions (including Fig. 7).

(3) RESPONSE: We understand the reviewers' concerns regarding our analyzing

parameters of the measured Ca2+ and Cl- signals. However, we are convinced that our study characterizes physiological relevant Ca2+ and Cl- dynamics. As described below, we had specific reasons for our experimental design and data analyses:

First, the odor stimulation of 2s and the recording time of 20s represent standard parameters that we and other labs normally use for calcium imaging recordings of odor-evoked responses in Drosophila.

Second, the frame rate of 2 Hz is of course low, but depends on the intrinsic fluorescence property and the signal-to-noise ratio of the fluorescent proteins used. Since Clomeleon yields a very low signal-to-noise ratio, we had to apply rather long exposure times which limited our recording frequency. We also performed experiments with the usually used frequency of 4Hz, resulting in weaker signal intensities and a lower dynamic range. Since these signals did not reveal different temporal patterns in the odor-evoked responses as the slower recorded signals and the responses are generally very slow, we decided in favor of

an increased signal-to-noise ratio and maintained a recording frequency of 2Hz for the whole study. Recording frequencies of fluorescence sensors with low signal-to-noise ratios, such

as Cameleon 2.1, GCaMP 1.6 or CaGreen are usually in the range of 2-3 Hz (e.g. Fiala et al., Curr. Biol, 2002; Galizia et al., Nat. Neurosci., 1999; Pelz et al., J. Neurobiol., 2006; Sachse et al., Eur. J. Neurosci. 1999; Silbering et al., J. Neurosci., 2008). Another option to decrease the exposure time and gain faster recording frequencies would be to apply a higher binning of pixels. However, since we were primarily interested in the spatial patterns of individual glomeruli of the odor evoked responses, a further decrease of spatial resolution as already used (i.e. fourfold binning with pixel resolution of 1.25 μm was applied) was not favorable.

Third, the reviewers are concerned about our delayed time window for signal evaluation. Although the chloride kinetics are clearly odor induced, they develop very slowly over time. Such long lasting chloride kinetics have also been shown in mice (Staley and Proctor, 1999, Berglund et al., 2009, Berglund et al., 2016) and are discussed in detail in our manuscript. In

addition, also the odor-evoked calcium responses show their maximal response after the end of the odor stimulus. Due to these long-lasting and slowly evolving Cl- and Ca2+- dependent responses, we chose a late time window for the response analysis in order to capture the maximum/minimum response, which occurs usually after the odor offset, and to improve the signal-to-noise ratio. Such analyzing parameters are not unusual for fluorescent sensors with low signal-to-noise ratios and have been applied for calcium imaging

recordings of odor-evoked responses in flies and bees where the maximum intensity change appears after the end of the odor stimulus (e.g. Pelz et al., J. Neurobiol., 2006; Sachse et

al., Eur. J. Neurosci., 1999). In addition, the used fluorescent protein Cameleon 2.1 has

slower temporal kinetics than non-ratiometric proteins as e.g. the GCaMP family (Strutz et al., 2012; Tian et al., Nat. Methods, 2009). One crucial problem could have been if the spatial (i.e. glomerular) response patterns of the odor-evoked Cl- and Ca2+-responses would change over the course of the long lasting responses. However, since this is not the case,

we are confident that using a late time window for signal evaluation reliably reflects the initial odor-induced changes and is therefore physiologically relevant. We agree that a detailed temporal comparison of Clomeleon with new indicators such as GCaMP6 has to be considered with caution and therefore selected the ratiometric sensor Cameleon 2.1, which shows comparable properties to Clomeleon, for our study.

Fourth, the Ca2+ and Cl- dynamics monitored are also dependent on the kinetics and

concentrations (i.e. expression levels) of the fluorescent sensors and might therefore not reflect accurately the physiological time traces. However, this issue is more relevant for fast stimulus dynamics as shown in Martelli and Fiala (eLife, 2019), while with regard to slow recording frequencies, as used in our study, the resulting kinetics of Cl- and Ca2+ binding are rather negligible.

To address the reviewers' concern, we have added two paragraphs in the method section explaining our analyzing strategy (pages: 6 & 8).

(4) REVIEWER: Minor comments: 1) line 165: Glomerulus “VM5” is traditionally divided into two glomeruli (VM5d and VM5v). This should be spelled out to avoid confusion. In addition, the authors describe VA6 as not labeled by GH146-Gal4, which is incorrect (Grabe et al,

2015, J Comp Neurol).

(4) RESPONSE: The reviewers are correct in mentioning that glomerulus VM5 is split into glomeruli VM5d and VM5v and that glomerulus VA6 is labeled by GH146-GAL4. In our study only glomerulus VM5v could be reliably identified and characterized at the OSN level. Unfortunately, we could not reliably identify glomeruli VM5d and VA6 and had to exclude

both from our analysis. We revised the corresponding sentence in the Material and Method

section (page 7).

(5) REVIEWER: 2) Line 203-204, and Figure 1: The biphasic responses of Clomeleon to GABA application should be interpreted in more detail. It would be interesting to have the authors' perspective on what causes this dynamics response to GABA.

(5) RESPONSE: We agree with the reviewers on the interesting fact of the delayed two-part GABA effect on the imaged PNs and considered diverse explanations for this effect. However, since the bath application procedure affects various cell types that are present in the antennal lobe (i.e. OSNs, LNs, PNs), it is very difficult to determine the exact mechanisms underlying these two phases. We assume that slow diffusion of GABA throughout the AL due to the bath application method leads to a gradual increase of the

GABA concentration that surpasses a certain threshold to initiate the second phase of stronger inhibition. In combination with a gradual desensitization towards GABA (Hong and Wilson, Neuron, 2015), it could explain the slow and biphasic GABA effect. We added this speculation to our manuscript (page 9).

(6) REVIEWER: 3) Lines 383-384 and Figure 7C,D: The authors should explain why (for both Cameleon and Clomeleon) the Euclidean distances are larger in ORNs than in PNs. Olsen et al (2010, Neuron) have reported that inhibition in the antennal lobe increases the discriminability of odors, i.e. PNs should have larger Euclidean distances than ORNs. Methodologies are different in important ways between these two studies, but it is important for the authors to explain why this discrepancy arises, as it may inform future studies.

(6) RESPONSE: Although PNs show an increased level of inhibition, PNs exhibit generally broader odor-evoked responses compared to OSNs (e.g. Wilson et al., Science, 2004; Seki et al., BMC Biol., 2017). This broadening leads to broader odor tuning curves and a stronger overlap of odor representations at the PN level (Niewalda et al., PLoSONE, 2011; Schubert et al., Front. Physiol., 2014; Seki et al., BMC Biol., 2017), which is in line with our results. Hence, PNs do not have necessarily higher odor discriminatory properties than OSNs, but in contrast show a higher degree of odor categorization according to behaviorally meaningful values, as e.g. hedonic valence (Knaden et al., Cell Rep., 2012; Niewalda et al., PLoSONE,

2011). We added this point to our result section (page 17).

(7) REVIEWER: 4) Lines 466-467: “widespread inhibition below baseline firing rates among

24...” This sentence is a bit misleading, as if the authors were suggesting that all 24 of the ORs tested by Hallem and Carlson (2006, Cell) conferred significant odor-specific suppression of firing rates upon their ORNs. A slight re-wording would help.

(7) RESPONSE: We agree and have revised the sentence to avoid confusion to “... widespread inhibition below baseline firing rates among one third out of 24 selectively ...” (page 21).
